# The role of serum calprotectin as a novel biomarker in inflammatory bowel diseases: a review study 

**Published:** 2019

**Authors:** Tayebeh Azramezani Kopi, Shabnam Shahrokh, Shahrokh Mirzaei, Hamid Asadzadeh Aghdaei, Azade Amini Kadijani

**Affiliations:** 1 *Student Research Committee, Babol University of Medical Sciences, Babol, Iran.*; 2 *Basic and Molecular Epidemiology of Gastrointestinal Disorders Research Center, Research Institute for Gastroenterology and Liver Diseases, Shahid Beheshti University of Medical Sciences, Tehran, Iran*; 3 *Bone and Joint Reconstruction Research Center, Shafa Orthopedic Hospital, Iran University of Medical Sciences, Tehran, IR Iran.*; 4 *Gastroenterology and Liver Diseases Research Center, Research Institute for Gastroenterology and Liver Diseases, Shahid Beheshti University of Medical Sciences, Tehran, Iran*

**Keywords:** Inflammatory bowel diseases, Serum calprotectin, Biomarker

## Abstract

As the incidence of inflammatory bowel diseases (IBD) increases in the newly industrialized countries, the health and financial burden of disease also increase. These changes make the role of IBD biomarkers further crucial. Serum calprotectin, as a novel blood-based biomarker of IBD, has been investigated in several investigations. Yet, there is no consensus regarding its clinical utility. We searched the electronic database, including PubMed, EMBASE, Scopus, and Web of Science up to the end of 2018 to find how serum calprotectin associates with the disease characteristics in IBD. The search terms included: inflammatory bowel diseases, IBD, Crohn Diseases (CD), Ulcerative Colitis (UC), calprotectin, serum, and blood. Based on our review, a biomarker role has been suggested for serum calprotectin in IBD, as significant associations were found between serum calprotectin and disease burden, prognosis, and relapse. A complementary role to fecal calprotectin has also been suggested for serum calprotectin. On the other hand, considering a significant correlation between serum calprotectin and serum CRP, but not fecal calprotectin, serum calprotectin could be more representative of systemic inflammation than an intestinal inflammation. Consequently, further researches are needed to unwrap the potential of serum calprotectin as a blood-based biomarker in IBD.

## Introduction

 Inflammatory bowel diseases (IBD), mainly ulcerative colitis (UC) and Crohn's disease (CD), are a group of disorders characterized by the prolonged inflammation of the gastrointestinal tract. Although the etiology of IBD is not well understood, environmental, genetic and immunologic factors have been considered as the major etiology of IBD. It is believed that IBD is the result of a dysregulated immune response to the host intestinal microflora in the genetically susceptible individuals (1, 2).

The incidence and prevalence of IBD are rapidly increasing in newly industrialized countries, supporting an association between the “westernization of lifestyles” and IBD (3). Accordingly, the Iranian incidence of IBD has revealed an increase from 0.62 to 3.11 per 100,000 from 1990 to 2012 (4). An annual percentage change of +4% for CD and +4.8% for UC has also been reported in Taiwanese population (3). This increased incidence highlights the necessity for more research into the prevention, diagnosis, and management of this complex and costly disease.

Currently, colonoscopy is the standard method for initial diagnosis, assessment of the disease burden, and evaluation of response to treatment (5). Even so, the colonoscopy is an invasive, expensive, and difficult-to-prepare patient method. Thus, the development of more convenient, inexpensive, and non-invasive assessments are crucial for IBD (6).

Laboratory biomarkers, as the noninvasive indices of disease assessment, can aid the management of IBD. To date, many laboratory biomarkers have been introduced for IBD (7-12). However, the majority of biomarkers currently in use are limited in their clinical utility to predict long-term outcomes of IBD. Serum markers of acute phase response such as C-reactive protein (CRP) and Erythrocyte Sedimentation Rate (ESR) have been widely investigated. However, they are not specific to IBD as their levels are also increased in various conditions such as infections, other autoimmune disorders, malignancy, etc (7). Serologic markers and antibodies such as Anti-neutrophil cytoplasmic antibodies (ANCAs) and anti-Saccharomyces cerevisiae antibodies (ASCA) are also of interest. Perinuclear ANCA is detected in 20–85% of UC and 2–28% of CD patients (13). ASCA is detected in 39–76% of CD patients, up to 15% of UC patients, and nearly 5% of healthy individuals (14). Amongst fecal biomarkers, fecal calprotectin is being routinely used in the management of IBD patients. However, an inconsistent sensitivity and specificity have been reported for fecal calprotectin in different investigations (15)‎. Moreover, in spite of the acceptable sensitivity and specificity of fecal calprotectin, very elderly patients might have difficulty with stool sample collection (16), and more feasible IBD biomarkers are still in demand.

Recently, serum calprotectin has been reported as a novel promising biomarker in monitoring IBD patients (17). Nevertheless, the value of serum calprotectin as a biomarker in IBD is controversial (18). In this review, we aimed to provide a comprehensive update on the value of serum calprotectin in the management of IBD. 

## Methods

We searched the electronic database including PubMed, EMBASE, Scopus, and Web of Science up to the end of 2018 to find how serum calprotectin associates with disease characteristics in IBD. The search terms included: inflammatory bowel diseases, IBD, Crohn Diseases (CD), Ulcerative Colitis (UC), calprotectin, serum, and blood. Searches were limited to humans and articles in English. The searches were not limited by publication type. Two authors (Tayebeh Azramezani kopi and Azade Amini Kadijani) independently extracted the data. 

**Immunobiology of Calprotectin** **‏**

Calprotectinis a calcium- and zinc-binding protein consisted of two small anionic proteins, S100A8, and S100A9. Its expression has been identified in the early differentiation stage of a variety of immune cells including macrophages, granulocytes, and ‎monocytes, following their activation by damage-associated molecular patterns (DAMPs) or pathogen-associated molecular patterns (PAMPs) (19, 20). Calprotectin contains direct bactericidal and pseudo-cytotoxic ‎mainly through the Mn2+ and Zn2+ chelation (21). Moreover, calprotectin is an endogenous ligand for toll-‎like receptor 4, which transfers the danger signal (20). Furthermore, calprotectin triggers ‎the inflammatory pathway upstream of the tumor necrosis factor α which is vital for lipopolysaccharides toxicity (22). ‎Calprotectin is also an effective mediator in the rapid rearrangement of tubulin-dependent cytoskeleton that is necessary for the cell ‎migration in polymorphonuclear leukocytes (23). Calprotectin plays a crucial role in phagocyte NADPH oxidase activation as well. So that in the presence of calcium, calprotectin‎ binds to the cytosolic arachidonic acid and transfer it to the nicotinamide adenine ‎dinucleotide phosphate oxidase complex in the neutrophil plasma membrane (24). ‎

After induction by lipopolysaccharides or chemokines, calprotectin expression upregulates in the neutrophils and will be translocated to the extracellular fluid. Then it triggers a variety of biological processes like signal transduction, cell homeostasis and inflammation (25). Calprotectin receptors with different affinities are identified at the surface of many immune cells. Attachment of these receptors to their ligands activates the inflammatory ‎signaling pathways such as nuclear factor-κB (NF-kB) and mitogen-activated protein kinase ‎‎(MAPK) pathway (26, 27). Calprotectin binds to the endothelial cells via Toll-like receptor 4 and carboxylatedglycans and triggers cell ‎activation (28). The calprotectin release also weakens the cell–‎cell contacts and as a result, the permeability of endothelium will be modified leading to the leukocyte ‎extravasation (29).

**Fecal calprotectin and IBD** **‎**

**Figure 1:**
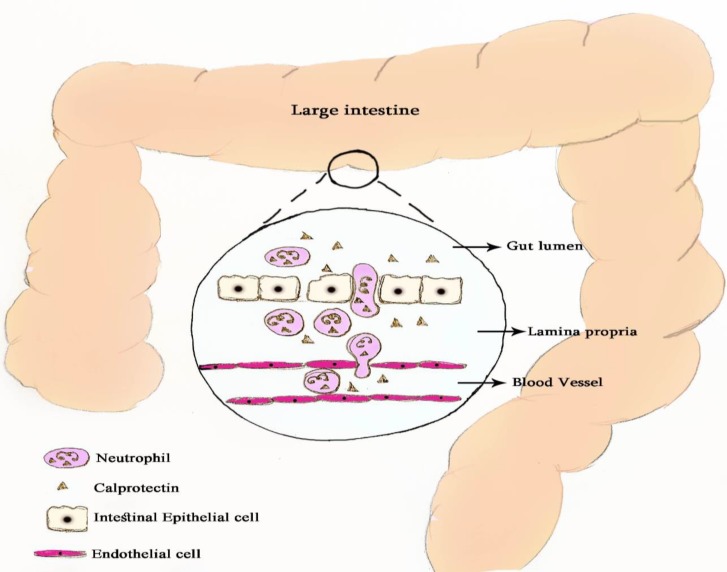
Schematic figure of calprotectin release into the gut lumen

Fecal biomarkers of gastrointestinal inflammation have attracted many attentions in the past decade. In this respect, fecal calprotectin has been widely studied as the most promising fecal biomarker of IBD. Following the gut inflammation, calprotectin is released by neutrophils influx into the intestinal lumen and then excreted in the stool, allowing the identification of gut inflammation (Figure 1). There is increasing evidence regarding the efficacy of fecal calprotectin in IBD monitoring, disease activity, and response to therapy as well as in predicting relapse (30).

Gisbert and McNicholl who combined data from 2,475 patients, obtained a mean sensitivity and specificity of 83% and 84% for fecal calprotectin respectively to distinguish organic from non-organic gastrointestinal disorders (31). Two consecutively elevated fecal calprotectin values have been reported to highly associated with IBD relapse (32). A significant correlation of as high as r=0.834 has been reported between fecal calprotectin and endoscopically assessed disease activity (33). In the study of D'Haens *et al.* fecal calprotectin of<250 µg/g was identified as mucosal healing with the sensitivity and specificity 94% and 62%, respectively. These findings suggest the application of fecal calprotectin in the evaluation of treatment efficacy (34). The study of Costa *et al.* revealed a 14-fold greater risk of relapse in UC patients with fecal calprotectin level of>150 µ/g, suggesting a prognostic role for fecal calprotectin (35). Many other investigations also have provided convincing evidence regarding the reliability of fecal calprotectin as a biomarker of diagnosis, relapse, prognosis, and response to treatment in IBD (36). 

**Serum calprotectin and IBD**

Recently, serum calprotectin has gained more attention as a biomarker for IBD as a blood-based biomarker may be more convenient in routine practice and more acceptable to patients. Kalla *et al.* evaluated the serum calprotectin level in a total of 156 patients (n=83 IBD, n=73 non-IBD) using multivariable logistic regression analysis. According to their results, serum calprotectin strongly correlated with fecal calprotectin and was the strongest predictor of IBD diagnosis in comparison with other biomarkers such as C-reactive protein (CRP) and albumin. Serum calprotectin was also capable of predicting treatment escalation and/or surgery in IBD patients, in particular, CD patients. Moreover, a model incorporating serum calprotectin and either CRP or albumin revealed a positive likelihood ratio of 24.14 for IBD. However, in a subset of 50 patients with paired serum and fecal calprotectin, fecal calprotectin was a better discriminating marker for the differentiation of IBD from controls. Kalla *et al.* concluded that a diagnostic and prognostic model with a combination of serum calprotectin and other blood-based biomarkers is capable of predicting the inflammatory burden in IBD patients, as well as to predict disease and its outcomes (37).

Hare *et al.* evaluated the diagnostic capability of serum and fecal calprotectin in 45 UC patients. Based on their results, serum calprotectin correlated significantly with CRP and with albumin but not with fecal calprotectin. A Receiver Operating Characteristic (ROC) curve analysis showed an area under the curve of 0.69 for serum calprotectin in comparison with 0.71 for CRP and 0.58 for fecal calprotectin. They concluded that further studies are needed to find if serum calprotectin could be a useful predictor of outcome in UC patients (38).

Meuwis *et al.* aimed to evaluate the value of serum calprotectin as a novel biomarker for CD in 115 patients in comparison with 40 healthy controls. Median serum calprotectin level was 8892 ng/mL in CD patients as in comparison with 1318 ng/mL in healthy controls. Serum calprotectin was significantly higher in active disease than the inactive disease. When serum calprotectin used as a discrete variable appeared complementary to high-sensitivity CRP and fecal calprotectin to predict CD relapse following the infliximab withdrawal. They concluded that serum calprotectin could be used as complementary to fecal calprotectin and high-sensitivity CRP to predict relapse after infliximab withdrawal (18).

Lügering *et al.* used a sandwich immunoassay to measure serum concentrations of calprotectin in 62 CD patients. Based on their results, serum calprotectin concentrations were significantly increased in patients with active CD when compared with patients with inactive CD. They concluded that serum calprotectin could be used for the discrimination of disease relapse (39).

Leach *et al.* assessed the role of serum calprotectin as a biomarker in a pediatric population with IBD. Based on their report, Serum calprotectin levels significantly correlated with the disease activity scores in children with IBD (40).

FuKunaga *et al.* evaluated the association between serum and fecal calprotectin in 41 patients with UC and in 13 patients with CD. They found no significant correlation between the serum and fecal calprotectin for either the UC or CD patients. A mild but significant correlation was found between the serum calprotectin and CRP levels in UC patients, but not in CD patients. According to this significant correlation, they concluded that serum calprotectin is likely derived predominantly from circulating leukocytes and not inflamed intestine. Hence, the serum calprotectin may reflect systemic inflammation, rather than gastrointestinal inflammation (17). 

In patients with ANCA-associated vasculitis, higher cell-surface calprotectin expression was recorded in circulating neutrophils and monocytes that were associated with increases in serum calprotectin levels. This finding further supports the derivation of serum calprotectin from circulating leukocytes and not diseased intestine (41).

McCann *et al.* investigated the performance of serum calprotectinas compared to the current biomarkers, FC, and CRP, in a cohort of 109 patients with GI disorders. They did not find a significant intraclass correlation coefficient between serum calprotectin, fecal calprotectin, and CRP. Accordingly, they concluded that as a blood-based biomarker for intestinal inflammation, serum calprotectin is unlikely to be of clinical utility (42).

**Table 1:** The role of serum calprotectin as a biomarker of IBD in different investigations

Author	Year	Disease	Patient number	Role as biomarker
Kalla *et al*. (37)	2016	IBD	35 CD, 45 UC, 3 IBDU	Disease burden, diagnosis, prognosis.
Hare *et al*.(38)	2013	UC	45	Prognosis
Meuwis *et al*.(18)	2013	CD	115	Complementary role to fecal calprotectin and hsCRP‎ in the prediction of relapse
Lügering *et al*.(39)‎	1995	CD	62	Discriminating the disease relapse
Leach *et al*. ‎(40)	2007	IBD	29 CD, 4 UV, 6 IBDU	Disease burden
Fukunaga *et al*. ‎(17)	2018	IBD	41 UC, 13 CD	Warrants further validation in large cohorts.
McCann *et al*. ‎(42)	2017	GI disorder	109	None

IBD: inflammatory bowel disease; UC: ulcerative colitis; CD: Crohn’s disease; IBDU: inflammatory bowel disease type unclassified; hsCRP: high sensitivity C-reactive protein.

Studies evaluating the role of serum calprotectin as a blood-based biomarker in IBD have been compared in Table 1.

The current study has some weak- and strong points. The small number of available publications on the role of serum calprotectin as a circulating biomarker in IBD was the main weakness of the present research which did not allow a comprehensive evaluation of the subject. By contrast, the present study is the only available publication reviewing the role of serum calprotectin as a biomarker of IBD, and this could be regarded as the strength of this study. 

## Conclusion

Serum calprotectin, as a novel blood-based biomarker for IBD, has been the subject of many recent investigations. However, there is no consensus regarding the role of serum calprotectin in the management of IBD. As most of earlier investigations have found a significant association between the serum calprotectin and CRP, but not fecal calprotectin. It is suggested that serum calprotectin may be more representative of systemic inflammation than an intestinal inflammation. However, further researches are needed to come to a conclusion in this regard, as the role of serum calprotectin in the prediction of relapse, determining the disease burden, and predicting the disease outcome in IBD have also been reported.

## Conflict of interests

The authors declare that they have no conflict of interest.

## References

[1] Wallace KL, Zheng LB, Kanazawa Y, Shih DQ (2014). Immunopathology of inflammatory bowel disease. World J gastroenterol.

[2] Cho JH (2008). The genetics and immunopathogenesis of inflammatory bowel disease. Nat Rev Immuno.

[3] Ng SC, Shi HY, Hamidi N, Underwood FE, Tang W, Benchimol EI (2017). Worldwide incidence and prevalence of inflammatory bowel disease in the 21st century: a systematic review of population-based studies. Lancet.

[4] Norouzinia M, Chaleshi V, Alinaghi S, Beheshti Shirazi SS, Keramatinia A, Nourian M (2018). Evaluation of IL-12A, IL-12B, IL-23A and IL-27 mRNA expression level genes in peripheral mononuclear cells of inflammatory bowel disease patients in an Iranian population. Gastroenterol Hepatol Bed Bench..

[5] Norouzinia M, Chaleshi V, Alizadeh AHM, Zali MR (2017). Biomarkers in inflammatory bowel diseases: insight into diagnosis, prognosis and treatment. Gastroenterol Hepatol Bed Bench..

[6] Falvey JD, Hoskin T, Meijer B, Ashcroft A, Walmsley R, Day AS (2015). Disease activity assessment in IBD: clinical indices and biomarkers fail to predict endoscopic remission. Inflammat Bowel Dis.

[7] Iskandar HN, Ciorba MA (2012). Biomarkers in inflammatory bowel disease: current practices and recent advances. Trans Res.

[8] Amini AK, Asadzadeh HA, Sorrentino D, Mirzaei A, Shahrokh S, Balaii H (2017). Transmembrane TNF-α Density, but not Soluble TNF-α Level, is Associated with Primary Response to Infliximab in Inflammatory Bowel Disease. Clin Trans Gastroenterol.

[9] Aghdaei HA, Kadijani AA, Sorrentino D, Mirzaei A, Shahrokh S, Balaii H (2018). An increased Bax/Bcl-2 ratio in circulating inflammatory cells predicts primary response to infliximab in inflammatory bowel disease patients. United Europ Gastroenterol J.

[10] Pardi D, Sandborn W (2005). Predicting relapse in patients with inflammatory bowel disease: what is the role of biomarkers?. Gut.

[11] Kadijani AA, Javadinia F, Gholamrezaei Z, Mirzaei A, Balaii H, Ghavami SB (2018). Apoptosis markers of circulating leukocytes are associated with the clinical course of inflammatory bowel disease. Gastroenterol Hepatol Bed Bench.

[12] Kadijani AA, Shahrokh S, Haghazali M, Aghdaei HA, Mirzaei A (2018). Inducible nitric oxide synthase as a potential blood-based biomarker in inflammatory bowel diseases. Gastroenterol Hepatol Bed Bench.

[13] Peyrin-Biroulet L, Standaert-Vitse A, Branche J, Chamaillard M (2007). IBD serological panels: facts and perspectives. Inflammatory bowel diseases..

[14] Reumaux D, Sendid B, Poulain D, Duthilleul P, Dewit O, Colombel JF (2003). Serological markers in inflammatory bowel diseases. Best practice & research Clinical gastroenterology.

[15] Van Rheenen PF, Van de Vijver E, Fidler V (2010). Faecal calprotectin for screening of patients with suspected inflammatory bowel disease: diagnostic meta-analysis. BMJ.

[16] Bjarnason I (2017). The use of fecal calprotectin in inflammatory bowel disease. Gastroenterol Hepatol.

[17] Fukunaga S, Kuwaki K, Mitsuyama K, Takedatsu H, Yoshioka S, Yamasaki H (2018). Detection of calprotectin in inflammatory bowel disease: Fecal and serum levels and immunohistochemical localization. Inter J Molecul Med.

[18] Meuwis MA, Vernier-Massouille G, Grimaud J, Bouhnik Y, Laharie D, Piver E (2013). Serum calprotectin as a biomarker for Crohn's disease. J Crohn's Colitis.

[19] Nacken W, Roth J, Sorg C, Kerkhoff C (2003). S100A9/S100A8: Myeloid representatives of the S100 protein family as prominent players in innate immunity. Microsco Res Techniq.

[20] Ehrchen JM, Sunderkötter C, Foell D, Vogl T, Roth J (2009). The endogenous Toll–like receptor 4 agonist S100A8/S100A9 (calprotectin) as innate amplifier of infection, autoimmunity, and cancer. J leukoc Biol.

[21] Sohnle PG, Hunter MJ, Hahn B, Chazin WJ (2000). Zinc-reversible antimicrobial activity of recombinant calprotectin (migration inhibitory factor—related proteins 8 and 14). J Infect Dis.

[22] Clark MA, Plank LD, Connolly AB, Streat SJ, Hill AA, Gupta R (1998). Effect of a chimeric antibody to tumor necrosis factor-alpha on cytokine and physiologic responses in patients with severe sepsis-a randomized, clinical trial. Critical Care Med.

[23] Vogl T, Ludwig S, Goebeler M, Strey A, Thorey IS, Reichelt R (2004). MRP8 and MRP14 control microtubule reorganization during transendothelial migration of phagocytes. blood.

[24] Kerkhoff C, Nacken W, Benedyk M, Dagher MC, Sopalla C, Doussiere J (2005). The arachidonic acid-binding protein S100A8/A9 promotes NADPH oxidase activation by interaction with p67 phox and Rac-2. FASEB J.

[25] Dhas DBB, Bhat BV, Gane DB (2012). Role of calprotectin in infection and inflammation. Current Pediatr Res.

[26] Björk P, Björk A, Vogl T, Stenström M, Liberg D, Olsson A (2009). Identification of human S100A9 as a novel target for treatment of autoimmune disease via binding to quinoline-3-carboxamides. PLoS Biol.

[27] Leclerc E, Fritz G, Vetter SW, Heizmann CW (2009). Binding of S100 proteins to RAGE: an update. Biochim Biophys Acta.

[28] obinson MJ, Tessier P, Poulsom R, Hogg N (2002). The S100 family heterodimer, MRP-8/14, binds with high affinity to heparin and heparan sulfate glycosaminoglycans on endothelial cells. J Biol Chem.

[29] Viemann D, Strey A, Janning A, Jurk K, Klimmek K, Vogl T (2005). Myeloid-related proteins 8 and 14 induce a specific inflammatory response in human microvascular endothelial cells. Blood.

[30] Mumolo MG, Bertani L, Ceccarelli L, Laino G, Di Fluri G, Albano E (2018). From bench to bedside: Fecal calprotectin in inflammatory bowel diseases clinical setting. World J Gastroenterol.

[31] isbert J, McNicholl A (2009). Questions and answers on the role of faecal calprotectin as a biological marker in inflammatory bowel disease. Dig Liver Dis.

[32] Heida A, Park K, van Rheenen PF (2017). Clinical utility of fecal calprotectin monitoring in asymptomatic patients with inflammatory bowel disease: a systematic review and practical guide. Inflammat Bowel Dis.

[33] Schoepfer AM, Beglinger C, Straumann A, Trummler M, Vavricka SR, Bruegger LE (2010). Fecal calprotectin correlates more closely with the Simple Endoscopic Score for Crohn's disease (SES-CD) than CRP, blood leukocytes, and the CDAI. Am J Gastroenterol.

[34] D'haens G, Ferrante M, Vermeire S, Baert F, Noman M, Moortgat L (2012). Fecal calprotectin is a surrogate marker for endoscopic lesions in inflammatory bowel disease. Inflammat Bowel Dis.

[35] Costa F, Mumolo M, Ceccarelli L, Bellini M, Romano M, Sterpi C (2005). Calprotectin is a stronger predictive marker of relapse in ulcerative colitis than in Crohn’s disease. Gut.

[36] Walsham NE, Sherwood RA (2016). Fecal calprotectin in inflammatory bowel disease. Clinical and ‎experimental gastroenterology..

[37] Kalla R, Kennedy NA, Ventham NT, Boyapati RK, Adams AT, Nimmo ER (2016). Serum calprotectin: a novel diagnostic and prognostic marker in inflammatory bowel diseases. Am J Gastroenterol.

[38] Hare N, Kennedy N, Kingstone K, Arnott I, Shand A, Palmer K (2013). PTH-082 Serum Calprotectin: A Novel Biomarker to Predict Outcome in Acute Severe Ulcerative Colitis?. Gut.

[39] Lügering N, Stoll R, Kucharzik T, Schmid KW, Rohlmann G, Burmeister G (1995). Immunohistochemical distribution and serum levels of the Ca2+-binding proteins MRP8, MRP14 and their heterodimeric form MRP8/14 in Crohn’s disease. Dig.

[40] Leach ST, Yang Z, Messina I, Song C, Geczy CL, Cunningham AM (2007). Serum and mucosal S100 proteins, calprotectin (S100A8/S100A9) and S100A12, are elevated at diagnosis in children with inflammatory bowel disease. Scandinavian J gastroenterol.

[41] Pepper RJ, Hamour S, Chavele KM, Todd SK, Rasmussen N, Flint S (2013). Leukocyte and serum S100A8/S100A9 expression reflects disease activity in ANCA-associated vasculitis and glomerulonephritis. Kidney Inter.

[42] McCann RK, Smith K, Gaya DR (2017). A prospective single centre pilot evaluation of a serum calprotectin assay in unselected GI patients. Clin Biochem.

